# Supplementary material for: In Vitro Study of the Multimodal Effect of Na+/K+ ATPase Blocker Ouabain on the Tumor Microenvironment and Malignant Cells
Source: Biomedicines. 2023 Aug 5;11(8):2205. doi: 10.3390/biomedicines11082205 (PMC10452365; doi:10.3390/biomedicines11082205)
Supplement: Supplementary file 1 [file biomedicines-11-02205-s001.zip › biomedicines-2441269-supplementary.pdf]

Supplementary materials

Immunocytochemistry of SK-BR-3 cells

Untreated and 24 hours Ouabain-treated SK-BR-3 cells were also used for showing expression of endoglin in immunocytochemistry testing. Monoclonal anti-human endoglin, CD105 (clone SN6h; Cat. no. M3527) was used in the staining protocol continued with secondary biotinylated antibody binding, substrate addition, and hematoxylin (Dako; Cat. no. CS70030-2) counterstaining of the nuclei (LSAB2 System-HRP, Cat. no. K0675 and Envision kit, Cat. no. K5007, Dako) following the manufacturer procedures. Microscopy visualization was performed on a Nikon Eclipse E800 microscope (Nikon, Tokyo, Japan). Endoglin marker was not expressed on SK-BR-3 tumor cells. Figure 1S shows lack of Endoglin marker, but reveals decreased adherent cell count in SK-BR-3 cells treated with 10^{-6} M Ouabain for 24 hours.

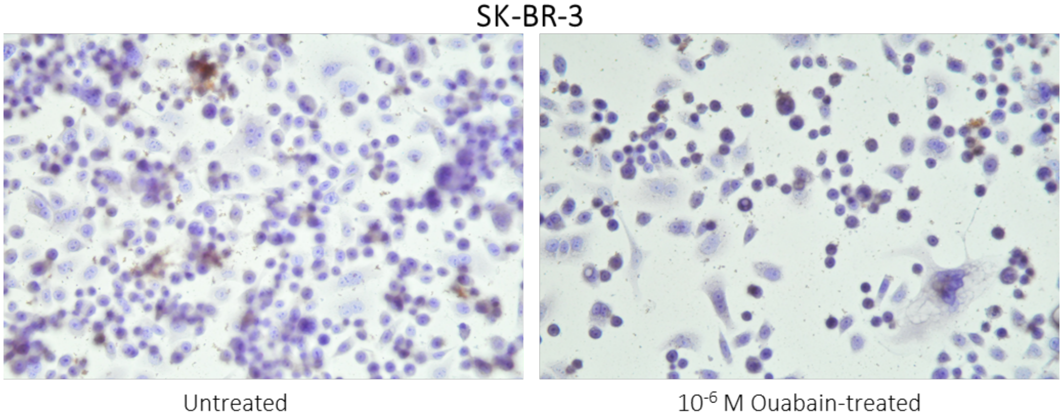

Figure S1. Endoglin marker is not expressed on SK-BR-3 cells. Tumor cells were exposed for 24 hours to 10^{-6} M Ouabain. Ob. 20x

MTT assay

In Vitro Toxicology Assay Kit, MTT based (Sigma-Aldrich Company, Cat. No. TOX1-1KT) was used for assessing the metabolic activity of cells. This assay is based on the reduction of MTT by cellular metabolism, and the formation of final product formazan is proportional to the metabolic activity of cells in culture.

All cell types (SK-BR-3, MSCs and TAFs) were seeded in quadruplicate into 96-well culture plates at the following cellular densities: 3 x 10^3, 5 x 10^3, and 10 x 10^3 cells/well. Cells were treated for 24 and 48 hours with Ouabain in concentrations of 10^{-5} M (C1), 10^{-6} M (C2), 10^{-8} M (C3), 10^{-9} M (C4). The values of % MTT reduction were corrected for background values of negative controls containing medium without cells. The absorbance was measured at 570 nm with a reference at 655 nm using a 96-well plate reader (BIO-RAD Microplate Reader, Benchmark). The viability rate was calculated according to the formula: cell viability (%) = (absorbance of the experimental samples/absorbance of the control) x 100 % (Figure 2S).

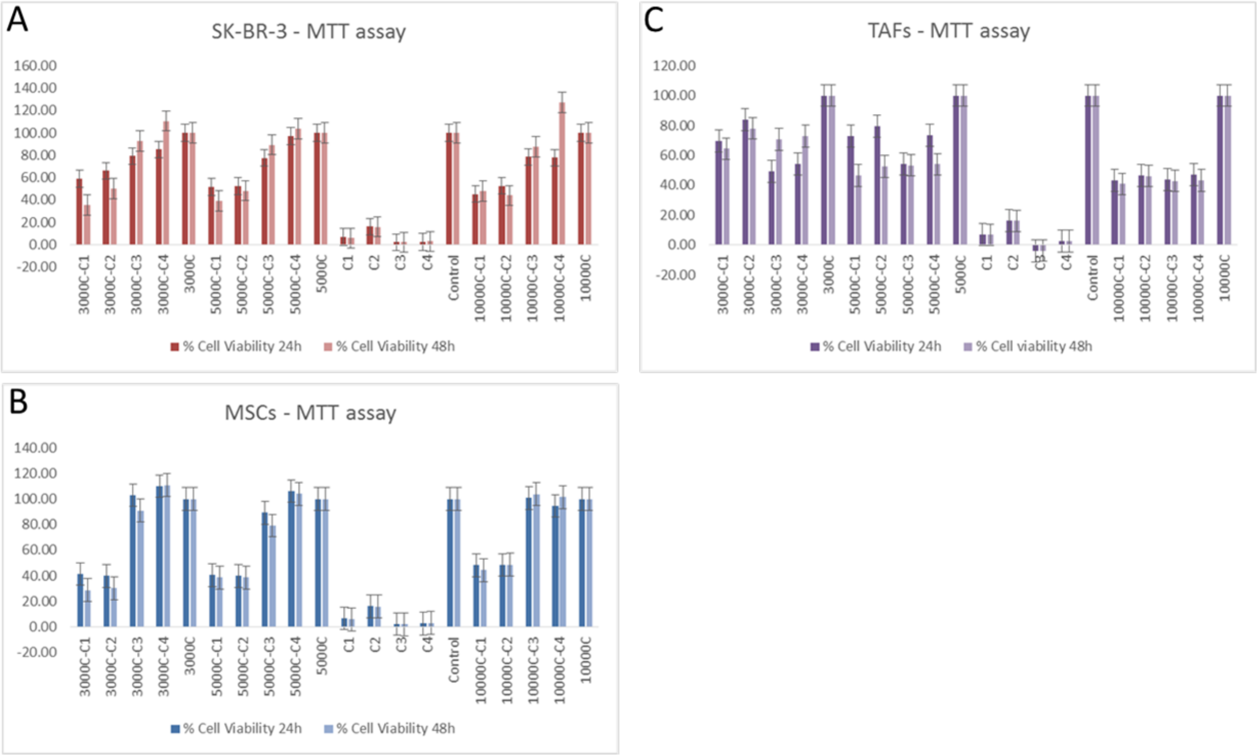

Figure S2. MTT assay at 24 and 48 hours after addition of Ouabain in the culture media. **A.** SK-BR-3 cells; **B.** MSCs; **C.** TAFs.

Legend:

*3000C = 3 x 10^3 cells/well; 5000C = 5 x 10^3 cells/well; 10000C = 10 x 10^3 cells/well

**C1-C4 are Ouabain concentrations: C1 = 10^{-5} M; C2 = 10^{-6} M; C3 = 10^{-8} M; C4 = 10^{-9} M

RT-PCR

RT-PCR reactions performed for Na^{+}/K^{+} ATPase α3, β2 and β3 subunits employed the primers presented in Tabel I (main manuscript) and visualization of the amplicons was accomplished in 1.5% agarose gels. Image J and Anlyze gels function was used for quantification and analysis of PCR agarose gels (Figure 3S).

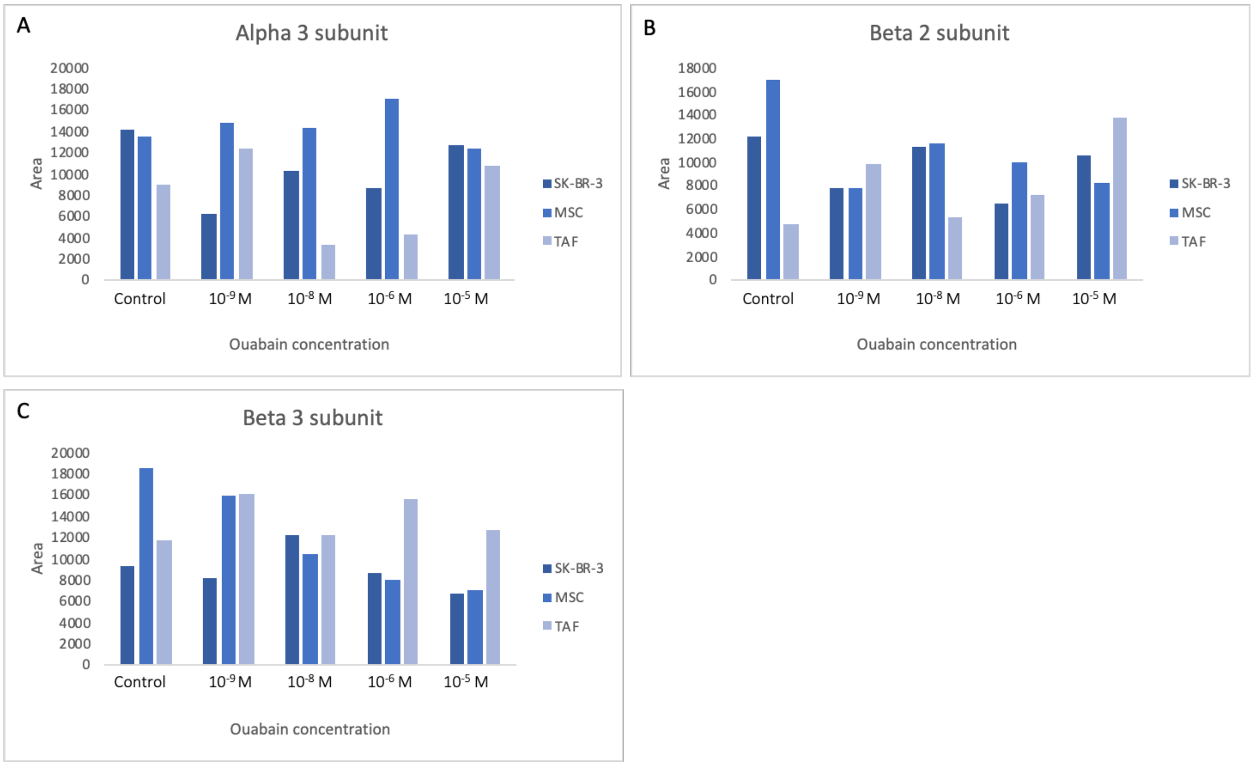

Figure S3. Quantification of RT-PCR gels for the Na^{+}/K^{+} ATPase subunits expression in SK-BR-3 cells, MSCs and TAFs in untreated (control) cells and 24 hours after addition in the culture media of Ouabain in different concentrations. **A.** α3 subunit expression in all cellular types, control and Ouabain-treated; **B.** β2 subunit expression; **C.** β3 subunit expression.
